# Psychometric Properties of the Turkish Version of the Coronavirus-Related Health Literacy Scale: Pinar Soylar

**DOI:** 10.1093/eurpub/ckac130.134

**Published:** 2022-10-25

**Authors:** P Soylar, F Öztürk, S Sönmez

**Affiliations:** 1 Health Science Faculty, Firat University, Elazig, Turkey; 2 Nursing Faculty, Ankara University, Ankara, Turkey; 3 Faculty of Education, Ege University, İzmir, Turkey

## Abstract

**Background:**

This study aims to assess the validity and reliability of the Coronavirus-Related Health Literacy Questionnaire adapted to Turkey and examine the levels of coronavirus-related health literacy among the adults.

**Methods:**

This cross-sectional study was carried out in Turkey. The tools were applied to a total sample population of 452 people. Exploratory Factor Analysis (EFA) and Confirmatory Factor Analysis (CFA) were calculated to determine the construct validity of the questionnaire with the IBM SPSS Amos program version 24.0.

**Results:**

The validity and reliability analyses of the Coronavirus-Related Health Literacy (HLS-COVID-Q22) questionnaire were adapted to Turkish. Internal consistency was very high (α = 0,963) and construct validity suggests a sufficient model fit, making HLS-COVID-Q19 a feasible tool for assessing coronavirus-related health literacy in population surveys. The findings show that the questionnaire is a valid and reliable tool consisting of 19 items and 3 subdivisions. The mean coronavirus-related health literacy score of the participants was found to be 2.92 (±0.51), meaning that it was on average. The coronavirus-related health literacy level of 18.8% of the participants was found to be ‘inadequate’ while 37.8% had ‘problematic’ and 43.4% ‘sufficient’ health literacy. The HLS-COVID-Q19-TR scores of those in the young age group (18-29 years old), married, employed, university graduates, and vaccinated against COVID-19 were found to be higher, and a statistically significant difference was found (p = 0.049, p = 0.009, p = 0.029, p = 0.012 and p = 0.051, respectively).

**Conclusions:**

The results of the research reveal that the HLS-COVID-Q19-TR is a valid and reliable tool. In this study, more than half of the participants were found to have “inadequate” or “problematic” coronavirus-related health literacy levels. For this reason, studies aimed at improving society's coronavirus-related health literacy should be conducted.

**Key messages:**

- There is now a valid tool to assess the Covid 19 health literacy level of individuals in Turkey. It will contribute to the determination of Covid 19 health literacy level in Turkey.
- An adequate level of health literacy helps people make informed decisions by combating misinformation about COVID-19 and supporting individuals’ self-management.

